# The Weak Worker Hypothesis: a new framework for understanding division of labour in social insects

**DOI:** 10.1111/brv.70068

**Published:** 2025-09-08

**Authors:** Jacob J. Herman, Alexander Walton, Olav Rueppell

**Affiliations:** ^1^ Department of Biological Sciences University of Alberta 116 ST and 83rd Ave Edmonton Alberta T6G 2E9 Canada; ^2^ Department of Biological Sciences Concordia University of Edmonton 7128 Ada Blvd NW Edmonton Alberta T5B 4E3 Canada

**Keywords:** division of labour, response threshold, social evolution, stress susceptibility

## Abstract

In social species, group functions often benefit from variation among individual group members. Many highly integrated social insect colonies rely on division of labour among colony members and emergent properties of their collective behaviour and physiology. Response threshold models are a prominent proximate explanation of division of labour, but how variation in response thresholds arise is largely unexplored. We propose the Weak Worker Hypothesis, a novel conceptual framework suggesting that response thresholds are determined by an individual's susceptibility to the stressor that underlies the task. Thus, specific tasks are preferentially performed, or at least initiated, by the individuals that are most susceptible to the corresponding stressor. Consequently, ‘weak’ workers that are susceptible to a particular stressor play a disproportionate role in the group's defence against this stressor. The response threshold manifests as an internal evaluation of a task‐specific stimulus that is influenced by the severity of the physiological perturbation of the individual, which simultaneously determines the susceptibility of this individual to succumb to the external disturbance. As long as individual stress susceptibilities vary among different stressors, this model generates division of labour and thus group stability. The Weak Worker Hypothesis provides a functional explanation for individual‐level responses to environmental deviations from optimal conditions. Such a deviation could be directly perceived as stimulus and simultaneously lead to physiological stress, or the physiological stress caused by the deviation could be the stimulus itself. In support of the Weak Worker Hypothesis, we present experimental evidence of a link between individual heat susceptibility and fanning behaviour in honey bees (*Apis mellifera* L.). We also discuss other possible cases and how to test our idea empirically in other contexts, keeping in mind the important distinction between cause and consequence. Finally, we conclude that the Weak Worker Hypothesis could provide a useful extension of response threshold models for understanding the division of labour in social groups, which might have repercussions for applied social insect science, selective breeding and eradication efforts.

## I. INTRODUCTION

The evolutionary and ecological success of social insects has been attributed to their cooperative behaviour, which allows them to construct elaborate nests, defend territories, and monopolize and rapidly exploit resources (Wilson, 1990). These advantages rely in large part on emergent properties of tightly integrated colonies, which are most apparent in the superorganismal eusocial insects (Wilson & Hölldobler, 2005; Hölldobler & Wilson, 2008; Boomsma & Gawne, 2018). Their colonial lifestyle allows for resource transfers among colony members and buffers individuals against environmental stress, providing protection to some individuals at the expense of others (A. Walton, J. J. Herman & O. Rueppell, 2024). These advantages rely on specialization of particular colony members to particular colony functions, resulting in division of labour (Bourke, 2011). Division of labour among individuals also provides key benefits to social insects by enhancing work flows and efficiencies through individual specialization (Leighton, Charbonneau & Dornhaus, 2017; Barrs *et al*., 2021).

Most fundamental to the evolution of insect eusociality is the reproductive division of labour (Simpson, 2012; Boomsma & Gawne, 2018), but division of labour is also found among the non‐reproductive workers of most social insects with respect to the vast majority of tasks. Many eusocial ants and termites have polyphenic worker castes with distinct developmental trajectories leading to permanent specialization (Wheeler, 1991; Wills *et al*., 2018; Pequeno, 2024). In species with uniform worker populations, an ontogenetic division of labour can occur, typically accompanied by age‐dependent physiological changes (Jeanson & Weidenmüller, 2014). Division of labour that is not based on age or morphology has often been attributed to other variability among individuals, such as genetic variation (Robinson & Page, 1988; Page & Robinson, 1991) or environmental influences before and during adulthood (Weidenmüller *et al*., 2009).

**Fig. 1:**
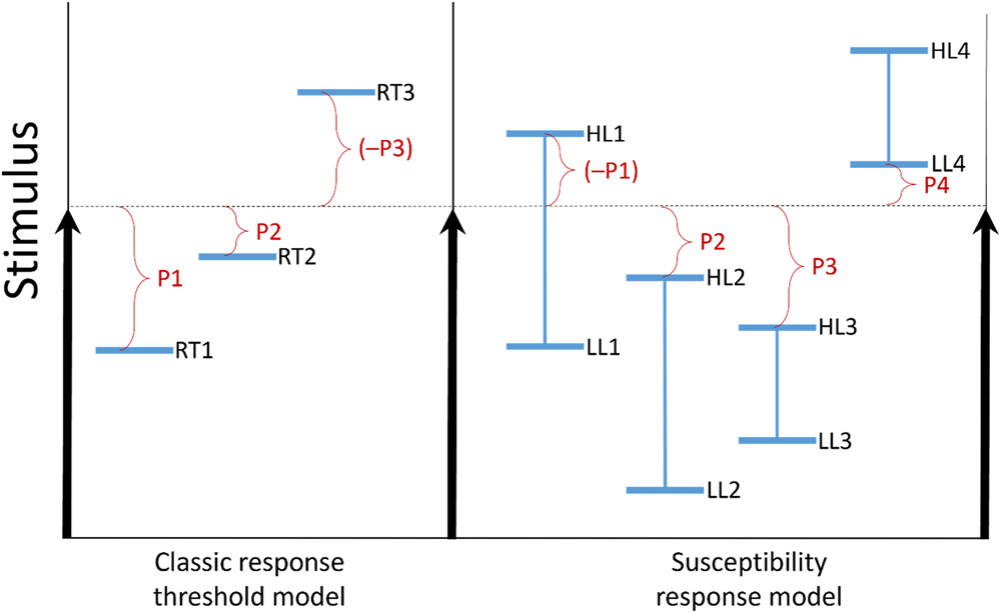
Comparison of the classical response threshold model compared to our susceptibility response model. Under the classical model (left), response thresholds of individuals are exceeded by a stimulus (dotted line), eliciting a response (RT1, RT2) or not (RT3). The distance of the stimulus from the response threshold (P1–P3) can be either interpreted as a response probability or the stimulus parameter space that elicits a response. By contrast, the Weak Worker Hypothesis suggests that response thresholds are better conceptualized as the relation between individual susceptibility to a stimulus/stressor and the stimulus level (right). Each of the four individuals has a tolerance interval ranging from a lower limit (LL1–LL4) to an upper limit (HL1–HL4). The stress is tolerated without a change in behaviour if the stimulus level (dotted line) is inside the tolerance interval (individual 1) but a behavioural response occurs if the stimulus level lies outside of this tolerance interval (individuals 2–4). The distance between the nearest limit of tolerance and stimulus level P1–P4) indicates either the response probability or parameter space, analogous to the classical model.

Even though some alternative explanations for the general organization of the division of labour in social insect colonies exist (Beshers & Fewell, 2001; Weidenmüller, Chen & Meyer, 2019), the response threshold model is most widely proposed as a mechanistic explanation of division of labour (Beshers, 2024). This model and its variations may be popular because the argument is simple, intuitive, and derived from principles of general animal physiology (Page & Mitchell, 1998). The response threshold model posits that individuals have different thresholds for task‐related stimuli and a stimulus that exceeds the threshold elicits a behavioural response (Fig. 1A). The resulting task performance in turn decreases the stimulus level leading to a homeostatic response *via* a negative feedback loop and the stimulus can be regarded as a perturbation or stressor (Robinson, 1987; Robinson & Page, 1988, 1989). At the colony level, the behavioural response will be proportional to the stimulus/perturbation if response thresholds are variable, with higher stimuli eliciting a response from more workers.

In specific cases, response thresholds have been empirically linked to behavioural specialization (Pankiw & Page, 2000; Han *et al*., 2021) and hormonal and neurophysiological modulators have been identified (Page & Erber, 2002; Amdam *et al*., 2006; Scheiner, Baumann & Blenau, 2006; Han *et al*., 2021). In general, however, response thresholds are poorly understood and, to our knowledge, a mechanistic explanation of response thresholds that involves a plausible biological basis and evolutionary pathway has not been adequately proposed. To address this deficit and stimulate more mechanistic research of response thresholds and behavioural specialization in social insects, we propose here that response thresholds are linked to individual stress susceptibility: more vulnerable individuals have a lower response threshold either through the direct perception of physiological stress or an evolved link between stress susceptibility and perception of and response to the corresponding stimulus. In either case a response threshold could be better understood as a certain level of stress that exceeds the tolerance limits of the individual (Fig. 1). Such links may have been vital for the survival of solitary individuals and thus likely pre‐date the evolution of sociality. In the context of social evolution, these links could have been co‐opted and further selected to diversify and govern division of labour. As explained in more detail below, we term this concept the ‘Weak Worker Hypothesis’ because the central adaptive argument is that individual weakness (i.e. susceptibility to a stressor) leads to division of labour and is therefore ultimately beneficial to the colony as a whole.

## II. THE WEAK WORKER HYPOTHESIS

Response thresholds have been a focus of study in social insects for decades (Robinson, 1987) but the concept pre‐dates social insect literature and can be traced to some of the first scientific explanations of innate behaviours through signal–response variation (Watson, 1930; Tinbergen, 1951). While there is extensive literature covering response threshold variation in other social insects, honey bees (*Apis mellifera* L.) have been studied in most detail. Various studies ascribe response threshold variation, and consequently division of labour, to a combination of age, genetics, and experience (Beshers, Robinson & Mittenthal, 1999; Pankiw & Page Jr, 2000). While proximate, neurobiological modulators of these factors have been identified in some cases (Page & Erber, 2002; Scheiner *et al*., 2006; Han *et al*., 2021), a more general explanation of why and how these factors influence response thresholds has not been proposed.

We suggest that age, genetics, and experience, potentially even including learning, influence response thresholds by generating variation in physiological stress susceptibility. Animal behaviour is deeply rooted in physiology and the perturbation of physiological homeostasis is thus a plausible candidate for a fundamental determinant of behavioural response to a stressor. Age‐related changes in physiology and task performance could be due to coordinated changes in specific stress susceptibilities and result in changing response thresholds. Genetic variation in stress susceptibility could also manifest as variation in response thresholds, where susceptibility arises from genetic influences, resulting in heritable variation in response thresholds that may act independently of age (Page, Erber & Fondrk, 1998; Lattorff & Moritz, 2013). Environmental influences and past experiences may also affect an individual's susceptibility to stress (Medina *et al*., 2020). Even apparent cases of reinforcement learning (Theraulaz, Bonabeau & Denuebourg, 1998) may have an alternative explanation if repeated performance of a task makes individuals more susceptible to the underlying stressor and thus decreases response thresholds without requiring learning to occur.

**Fig. 2:**
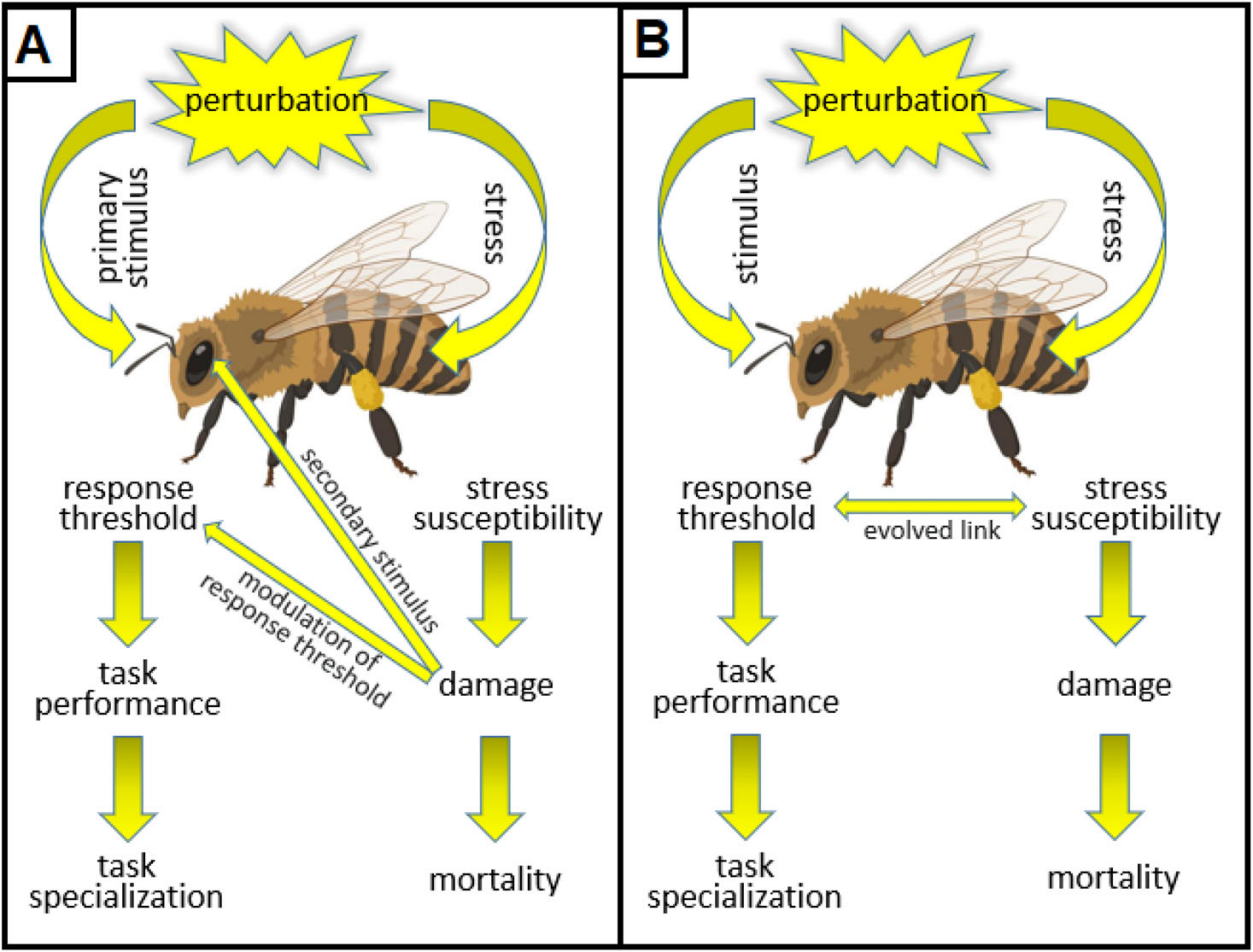
Models of possible links between stress susceptibility and response threshold. (A) The response threshold to a stress‐related stimulus could be modulated by physiological damage. The amount of damage is influenced by the individual's stress susceptibility, thus generating an indirect link. Alternatively, physiological damage could also act as a secondary stimulus that triggers task performance. (B) A direct link between an individual's stress susceptibility and response threshold to a corresponding stimulus may have evolved in solitary ancestors to trigger a protective behavioural response and prevent physiological damage. This evolved link could then be co‐opted by social evolution to optimize colony homeostasis by organizing division of labour.

A link between stress susceptibility and behaviour is likely not novel to social insects and may first have evolved in their solitary ancestors and thus might have ancient origins (Stefano *et al*., 2002). Three non‐mutually exclusive models to explain a link between a stressor and an organism's stress response are conceivable (Fig. 2). A behavioural response could arise directly in response to physiological damage created by the stressor. Under this first model, the physiological effects of the stressor would be a secondary stimulus, causing a behavioural response when the internal physiological buffering capacity is exceeded (Fig. 2A). The second model entails modulation of an individual's response threshold by physiological damage. This model is more permissive because it allows response thresholds to be determined by a variety of factors that can then interact with stress effects, but both of these models postulate direct stress effects (Fig. 2A). Workers that experience damage at low levels of a stressor (the ‘weak workers’) are predicted to be the first to respond to these low stress levels because they are the only ones suffering damage. Alternatively, if certain stimuli predictably lead to a corresponding physiological stress, an evolutionary link between behavioural responsiveness and stress susceptibility is favoured as an adaptation to pre‐empt physiological damage by behavioural responses (Fig. 2B). In this model, the ‘weak workers’ are predicted to have evolved a low behavioural response threshold to the stimulus and are more likely to perform tasks to decrease the stimulus and corresponding stressor.

Genetic diversity has repeatedly been demonstrated to benefit colony performance (Graham *et al*., 2006; Mattila & Seeley, 2007; Tarpy, vanEngelsdorp & Pettis, 2013; Desai & Currie, 2015), which can at least in part be attributed to enhanced division of labour when response thresholds have a genetic basis (Fewell & Page Jr, 1993; Beshers & Fewell, 2001; Gove *et al*., 2009). Natural selection for colony‐level phenotypes thus often involves selection for variation in individual response thresholds. According to our model, we predict selection for variation in individual stress susceptibilities. To the extent that response thresholds for different tasks need to vary independently, we also predict that selection favours stress susceptibilities to vary independently of each other. This prediction could be tested by comparing the degree of cross protection to different stressors among colony members with that among individuals of solitary species. More generally, we suggest an experimental framework to investigate the Weak Worker Hypothesis by testing its central prediction that individual susceptibility (‘weakness’) to a particular stressor is directly related to the likelihood of performing behaviours that reduce this stress at the colony level. In this simple two‐step experimental design (*i*) individuals participating in colony‐level stress response behaviours are collected along with non‐responders that can serve as an appropriate control group, and (*ii*) the two groups are subsequently compared for individual susceptibility to the corresponding stressor (Table 1). In this and other testing paradigms of the Weak Worker Hypothesis, it is crucial to distinguish cause from consequence. Thus, the reasons for performing a behaviour cannot be confounded with the effects of performing the behaviour. While consequences of a behaviour may reinforce a behavioural phenotype, particularly for persistent specialization such as foraging, the initial causation needs to be considered separately. In the following section, we report our findings from a test case of this experimental design in which we interrogate the Weak Worker Hypothesis as it applies to heat stress.

**Table 1:** Predictions of the Weak Worker Hypothesis by stressor. CT_max_, maximum critical temperature; CT_min_, minimum critical temperature.

Stressor	Colony response	Potential individual measurements	Prediction of Weak Worker Hypothesis	Current evidence
**Heat**	Fanning	CT_max_ and time to death	Fanning bees will die more quickly at higher temperatures	This study
**Cold**	Clustering thermogenesis	CT_min_ and cold coma recovery	Bees initiating clustering and heating cells will be more susceptible to cold	Free & Spencer‐Booth (1960); Stabentheiner *et al*. (2003)
**Pathogens**	Hygienic behaviour	Infection tolerance	Bees that participate in hygienic behaviour will have low infection tolerance	?
**Food**	Collective food gathering and storage	Starvation tolerance	Foragers will die of starvation before other workers	Remolina *et al*. (2007); Speth *et al*. (2015)
**Toxins**	Pollen entombing	Toxin tolerance	Bees participating in pollen entombing will have the lowest toxin tolerance	?

## III. TEST CASE: THERMAL TOLERANCE OF FANNERS

As an initial evaluation of the Weak Worker Hypothesis, we compared individual thermal tolerance between workers that engaged in fanning behaviour and control workers. Thus, we applied our suggested experimental paradigm of pairing an induced colony‐level stress response assay with an individual stress‐tolerance challenge. As predicted by the Weak Worker Hypothesis, we found that the first bees to initiate fanning behaviour had the lowest individual heat tolerance.

### (1) Methods

Honey bee (*Apis mellifera* L.) colonies were maintained at the Rueppell Laboratory Apiary at the Laird W. McElroy Metabolism and Environment Research Unit on the University of Alberta's South Campus in Edmonton, AB, Canada (53°30′10.5″N 113°32′13.6″W). Experimental colonies (*N* = 2) were queenright, five‐frame Langstroth‐style plastic EZ Nuc hives (Jester Bee Co.) with queens from local Alberta beekeepers. The experiment was performed in August 2023.

**Fig. 3:**
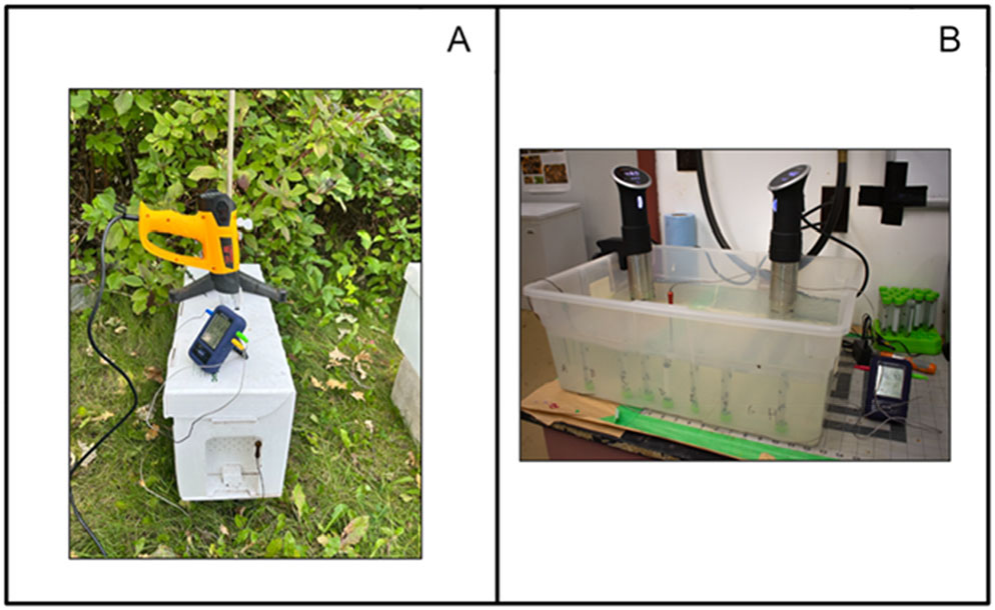
Methods used to investigate thermal tolerance of fanners. (A) Experimental setup to induce fanning behaviour. A heat gun delivers hot air into an EZ Nuc hive while thermal sensors record the temperature at the hive entrance and in an opening at the top of the hive. (B) Experimental setup to test individual thermal tolerance. Individual bees in 15 ml centrifuge tubes are submerged in a heat bath. Two heater‐circulators maintain the water temperature at 48 °C.

To induce fanning behaviour, we artificially raised the temperature of experimental hives by placing the nozzle of a heat gun (K Kernowo) in an opening at the top of the hive. Thermal probes (ENZOO Wireless Food Thermometer) recorded temperatures at the entrance of the hive and at the top of the hive beside the heat gun nozzle (Fig. 3A). The heat gun continuously delivered 45 °C hot air into the hive, a temperature shown to induce a colony‐level thermoregulatory response in honey bees (Jhawar *et al*., 2023). The heat gun raised the internal temperature from 29 °C to 34 °C in 4.5 min (the temperature and time when we first observed fanning behaviour at the hive entrance). Bees were identified as fanners if they were situated at the hive entrance, oriented with head facing the hive, wings rapidly fluttering, and abdomen raised, but without the Nasonov gland exposed (Cook & Breed, 2013). We removed fanners (*N* = 42) with soft forceps and placed them in a Plexiglas cage, sorted by home colony, in addition to bees collected without known age matching, a subset of bees of known age (14 days old, *N* = 12) were collected if fanning. Next, we removed control ‘non‐fanner’ bees from inside the hive that were situated near the entrance but were not fanning (*N* = 41) and sorted them into cages by home colony. Cages with bees were kept in an incubator at 33 °C and provisioned with *ad‐libitum* 50% sucrose solution overnight.

To test the individual thermal tolerance of fanners and non‐fanners, we constructed a hot water bath using a large plastic bin filled with water, two sous vide water heater‐circulators (Anova A3.2‐120V‐US Precision Cooker 900 W 110–120 V‐ac), and two thermal probes (Fig. 3B). 24 h after fanners and non‐fanners were collected from hives, we removed them from the incubator, placed them in individual 15 ml centrifuge tubes (uniquely labelled but keeping experimenters blind to the bees' behaviour category), and submerged them in the water bath at 48 °C. This temperature was chosen based on results of preliminary tests of our experimental apparatus. The tubes were submerged by pairing magnets in the tube caps with magnets underneath the water bath. We recorded time from initial submersion until death when movement completely ceased for each individual worker.

Survival of fanners and non‐fanners were compared using a linear mixed‐effects model with the ‘lmer’ function in the R package *lme4* (Bates *et al*., 2015) within R v. 4.3.1 (R Core Team, 2023), accounting for hive origin as a random effect.

### (2) Results and discussion

**Fig. 4:**
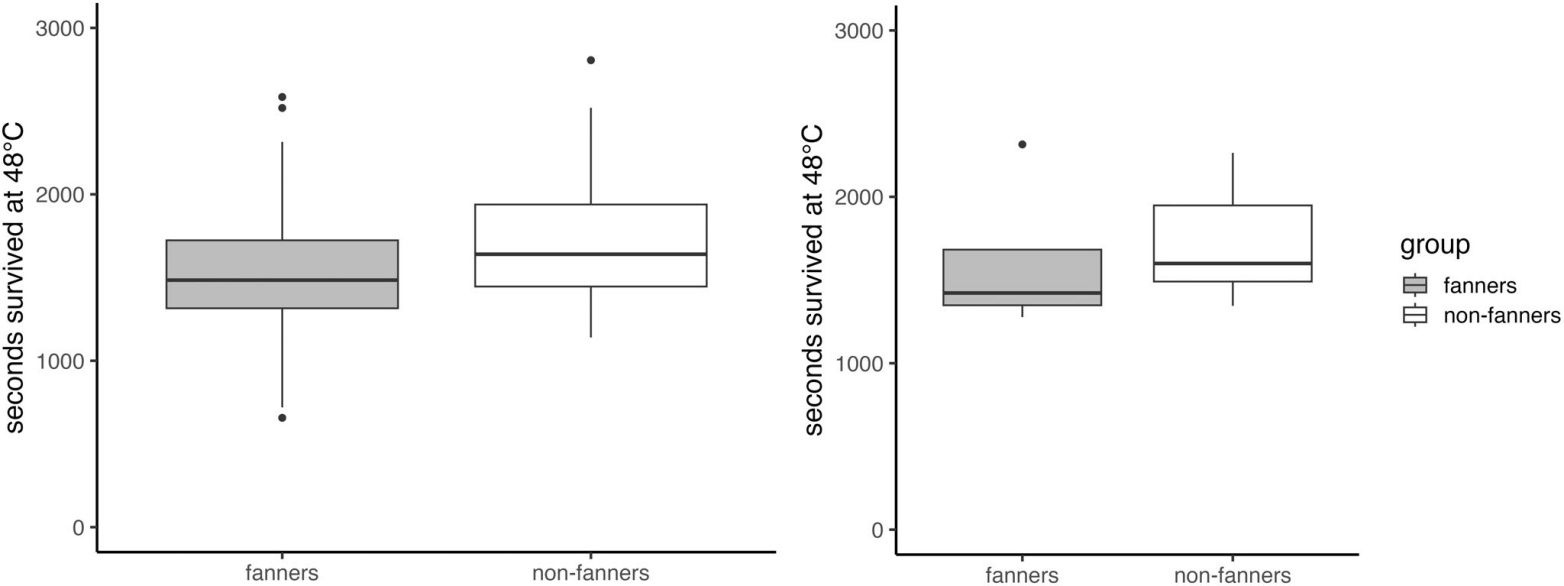
Boxplots of heating experiments. Left graph represents seconds survived in heat bath for the subset of age‐matched bees separated into fanning and non‐fanning bees. Right graph represents seconds survived in heat bath of the entire data set separated into fanning and non‐fanning bees.

When held at a lethal temperature of 48 °C, bees that initiated fanning died significantly earlier than the non‐fanning bees collected at hive entrances (linear mixed‐effects model: *t* = 2.61; df = 2.08, 21.15; *P* = 0.02; *N* = 42 fanners, 41 non‐fanners; Fig. 4 and Table S1).

We found initial empirical support for the Weak Worker Hypothesis by testing the individual thermal tolerance of bees that engaged in fanning behaviour, a key colony‐level stress response to high heat. By sampling the fanners at the onset of their behavioural response, we minimized the potential reverse causal explanation that fanning behaviour results in lowered heat stress resistance, although we cannot completely exclude this possibility. A more rigorous test would also include more age‐matched bees, larger sample sizes, and more colonies; these initial results are therefore only preliminary. Our study demonstrates that the Weak Worker Hypothesis provides testable predictions for the relationship between behavioural response thresholds and individual stress tolerance.

## IV. SCOPE OF THE WEAK WORKER HYPOTHESIS

Although we propose the Weak Worker Hypothesis as a general framework that connects susceptibility to an underlying stress stimulus to a behavioural response, we acknowledge that this hypothesis may not explain all behavioural response thresholds in social insects. For example, the Weak Worker Hypothesis may not apply to individuals that guard and/or attack intruders, since these individuals need to have morphological adaptations to perform defence behaviours. The Weak Worker Hypothesis also could potentially fail to explain hygienic behaviour response thresholds in honey bees if performing hygienic behaviour necessitates a higher rather than lower tolerance to pathogen exposure resulting from handling and removing dead or diseased brood (Posada‐Florez *et al*., 2021). Direct testing of the pathogen susceptibility of bees that perform hygienic behaviour will be necessary to determine if the Weak Worker Hypothesis can explain response threshold variation for this and other social immunity behaviours. In general, the Weak Worker Hypothesis may be less applicable for tasks that involve communication among castes such as hygienic behaviour (Wagoner *et al*., 2020), because signalling is likely to decouple the stressor and task. We suggest that the Weak Worker Hypothesis should be tested for each specific task, and could not only improve our understanding of division of labour in social insects but also clarify the origins of response thresholds.

To determine the generality of the Weak Worker Hypothesis, it will be necessary to test a variety of species and contexts. In Table 1, we list potential stressors that could be further investigated in social insects using our experimental framework. Although our thermal tolerance study is the first to test our new hypothesis directly, some previous studies that have measured individual‐ and colony‐level responses offer indirect tests of the hypothesis (Table 1). For example, previous research on honey bee cold tolerance suggests that individuals who initiate thermogenesis in winter clusters are particularly unable to withstand cold temperatures outside the cluster (Free & Spencer‐Booth, 1960; Stabentheiner *et al*., 2003). Future studies could collect bees performing brood/colony heating and test them for resilience to prolonged cold temperatures. The Weak Worker Hypothesis predicts that bees performing thermogenesis in winter clusters will have the highest minimum critical temperature (CT_min_) when tested for individual cold tolerance. The hypothesis further implies that individuals with poor cold tolerance are essential to colony thermogenesis. This would have implications for cold‐weather beekeeping practices through choices in starting bees for overwintering and for social insect overwintering models.

Pathogen stress has been tested at the colony and individual levels separately but not together in a single study. One experimental design could involve collection of bees performing hygienic behaviour and inoculation with a virus followed by individual scoring for survival outside of the colony. A relatively high susceptibility to pathogens of bees that perform hygienic behaviour would support the Weak Worker Hypothesis. Such a finding would appear counterintuitive (i.e. hygienic bees could be expected to have a higher tolerance to pathogens), but may be partially supported by some published evidence (Harpur *et al*., 2014). Hygienic behaviour is a particularly intriguing case where weaker workers might not necessarily be adaptive. It could perhaps explain task specialization, with particularly responsive workers responsible for uncapping of diseased brood cells, but less‐responsive and less‐susceptible workers actually removing the diseased brood (Barrs *et al*., 2021). If true, this would have implications for honey bee health research, suggesting the importance of maintaining a diversity of individual susceptibility to pathogens in colonies to ensure effective hygienic behaviour to reduce disease at the colony level (Erez *et al*., 2022). Breeding efforts to maximize individual pathogen tolerance therefore may not be sufficient to maintain colony‐level health.

Starvation is a stressor that affects both individuals and colonies and may offer the strongest evidence for a link between susceptibility and behaviour. It may also be a case where the ancestral link between hunger and foraging behaviour is most intuitive (Lin, Senapati & Tsao, 2019). Foragers are known to be some of the least starvation‐tolerant individuals in the colony (Remolina *et al*., 2007; Speth *et al*., 2015; Walton & Toth, 2023), and actively perform behaviours that increase colony‐level food stores. Further, nurse‐age bees that undergo starvation and manipulated depletion of fat stores have been shown to convert precociously into foragers and this pathway is separate from the socially regulated onset of foraging (Toth *et al*., 2005). This evidence supports individual starvation state being directly linked to the performance of behaviours to increase colony food stores.

Toxins represent a broad stressor category; many chemicals can be toxic, dependent on dose. While the lethal effects of many pesticides have been tested at the individual level for honey bees (Johnson, 2015; Farruggia *et al*., 2022), no study has yet related variation in individual susceptibility to colony performance or survival. Behavioural paradigms may need to be developed to test behavioural responses to toxin stress accurately, but we consider pollen entombing a possible colony‐level regulation of toxin stress that could be related to individual susceptibility to the corresponding chemicals (Table 1). Other experimental designs may be conceivable for specific chemical toxins.

While individual honey bees may initiate a task in direct response to a stress stimulus, others perform behaviours as a result of recruitment. A well‐known example of recruitment in honey bees is the waggle dance performed by forager bees to induce others to initiate foraging (Seeley, 1983). Another example of recruitment is fanning behaviour, which can be triggered both by increasing temperature and by the presence of other fanning individuals (Kaspar, Cook & Breed, 2018). To incorporate recruitment into the Weak Worker Hypothesis, the first responders to a stressor may serve as the colony's detection system and could be thought of as ‘stress scouts’. A different explanation may be that first responders represent specialists – the most susceptible of an already susceptible group. Individuals that respond to indirect stimuli (recruits) may represent transitional workers with flexible responses to multiple stressors. Future studies that combine large ranges of susceptibility of workers within an age/task group could help elucidate differences between stress‐induced and recruitment‐induced behaviours.

The Weak Worker Hypothesis predicts intra‐colonial variation in stress susceptibility to be adaptive. Thus, social evolution may have relaxed selection for individual stress‐resistance mechanisms that are present in solitary species (Dimopoulos *et al*., 2002; Girardot, Monnier & Tricoire, 2004; Guo, Jiang & Xia, 2016) or selected for modifiers or condition dependence of these mechanisms. Signatures of this selection could be detected by comparing genomes across the spectrum of sociality (Kapheim *et al*., 2015; Mikhailova, Rinke & Harrison, 2024), but the plasticity of stress defence mechanisms can also be experimentally compared between solitary and social species. In queens and drones, which are subject to radically different selection pressures, caste‐specific gene expression could drive susceptibility variation. Future studies that associate gene expression with caste‐specific behaviour could examine if gene expression profiles overlap with both increased *and* decreased expression of homologous genes in solitary organisms when challenged by the same stressors.

The Weak Worker Hypothesis may also have repercussions for practical apicultural breeding. Honey bee breeding has largely focused on selection for colony‐level traits (Rueppell, 2014; Niño & Jasper, 2015). Such programs have focused on reducing aggression and absconding, increasing honey production and overwintering survival, and decreasing disease through hygienic behaviour (Bar‐Cohen, Alpern & Bar‐Anan, 1978; Breed, Guzmán‐Novoa & Hunt, 2004; Hepburn, 2011; Danka, Harris & Dodds, 2016; Holmes *et al*., 2023). In addition to these breeding efforts, some individual‐level traits have been directly selected, such as queen morphology and behaviour (Hatjina *et al*., 2014; Facchini *et al*., 2021). While such breeding efforts have occurred since the earliest husbandry of honey bees, other traits have received more recent attention in light of the ongoing honey bee health crisis. For most traits, colony‐level selection dominates practical beekeeper practices and targeted breeding efforts alike (Rinderer *et al*., 2010). However, the relationship between colony‐level and individual‐level traits remains unclear. This is particularly important as future breeding practices may involve marker‐assisted selection (Sainsbury *et al*., 2022) and these markers can be identified in individual assays (Behrens *et al*., 2011). The Weak Worker Hypothesis argues that selection for variation in stress susceptibility might be more successful than unidirectional selection for higher stress resistance. A fraction of susceptible workers could be important to regulate and perform colony‐level defences. Colony health might even be improved by mixing stocks of varying stress susceptibilities. These ideas need to be tested, but there are intriguing possibilities, such as investigating the individual disease susceptibility of resin‐collecting workers that generate propolis as an immunity defence for the colony (Simone, Evans & Spivak, 2009). We caution against practices that rely on individual‐level stress resistance and selection based on individual traits of reproductive castes.

This discussion has relied heavily on honey bees, one of many social insect species in which there is age polyethism as organizing principle of division of labour. According to the Weak Worker Hypothesis, age polyethism will occur primarily due to physiological changes that alter stress resistance throughout the life of a worker. However, division of labour is organized by morphologically distinct worker subcastes in numerous other social insect species. These species have permanent and, in some cases, highly variable worker phenotypes, which should provide powerful tests of the Weak Worker Hypothesis. Permanent differentiation, allometry, and size dependency of many biological functions could lead to even greater variation in individual stress susceptibility than in species with monomorphic worker populations. A more direct relationship between stress stimuli and task performance can be expected in individuals that permanently engage in a limited number of tasks throughout their lives. Well‐studied social insects with morphological polyethism (bumble bees, fire ants, termites, leafcutter ants, etc.) would be suitable test subjects, especially in systems where individual‐ and colony‐level stressor assays already exist (Bujan *et al*., 2020; Arango *et al*., 2021; Quinlan *et al*., 2023).

## V. CONCLUSIONS

(1) The Weak Worker Hypothesis presents a novel concept to explain regulation of division of labour in social insects.
(2) We may need to rethink the causation and evolution of behavioural response thresholds.
(3) The hypothesis may have practical implications for honey bee health and breeding.
(4) We hope to stimulate a new generation of empirical studies on response thresholds and division of labour under this perspective.

## AUTHOR CONTRIBUTIONS

J. J. H conceptualized the initial ideas. J. J. H and A. W. designed and performed the heating experiments. J. J. H, A. W. and O. R. all participated in writing the final manuscript.

## Supporting information


**Table S1.** Heat bath experimental data.
