# Supplementary material for: The Weak Worker Hypothesis: a new framework for understanding division of labour in social insects
Source: Biol Rev Camb Philos Soc. 2025 Sep 8;101(1):5–13. doi: 10.1111/brv.70068 (PMC12783416; doi:10.1111/brv.70068)
Supplement: Supplementary file 1 — Table S1. Heat bath experimental data. [file BRV-101-5-s001.docx]

**Table S1.** Heat bath experimental data.

| **Trial** | **Group** | **Time to death (seconds)** | **Age group** | **Date** |
| --- | --- | --- | --- | --- |
| 2.1 | fan | 657 | unknown | 9/26/2024 |
| 0.1 | fan | 720 | unknown | 9/23/2024 |
| 0.1 | fan | 900 | unknown | 9/23/2024 |
| 0.1 | fan | 960 | unknown | 9/23/2024 |
| 2.2 | fan | 1027 | unknown | 9/26/2024 |
| 0.1 | fan | 1080 | unknown | 9/23/2024 |
| 2.1 | fan | 1092 | unknown | 9/26/2024 |
| 0.1 | nonfan | 1140 | unknown | 9/23/2024 |
| 1.2 | nonfan | 1173 | unknown | 9/24/2024 |
| 0.1 | fan | 1200 | unknown | 9/23/2024 |
| 2.1 | nonfan | 1219 | unknown | 9/26/2024 |
| 2.2 | nonfan | 1252 | unknown | 9/26/2024 |
| 2.1 | fan | 1277 | 14 days | 9/26/2024 |
| 2.2 | fan | 1280 | unknown | 9/26/2024 |
| 2.2 | fan | 1314 | unknown | 9/26/2024 |
| 0.1 | fan | 1320 | unknown | 9/23/2024 |
| 0.2 | fan | 1320 | unknown | 9/23/2024 |
| 2.1 | fan | 1331 | unknown | 9/26/2024 |
| 2.1 | nonfan | 1345 | 14 days | 9/26/2024 |
| 1.1 | nonfan | 1360 | unknown | 9/24/2024 |
| 2.2 | fan | 1372 | 14 days | 9/26/2024 |
| 0.2 | fan | 1380 | unknown | 9/23/2024 |
| 1.1 | nonfan | 1398 | unknown | 9/24/2024 |
| 2.2 | fan | 1410 | unknown | 9/26/2024 |
| 1.1 | nonfan | 1424 | unknown | 9/24/2024 |
| 1.2 | fan | 1428 | unknown | 9/24/2024 |
| 0.2 | nonfan | 1440 | unknown | 9/23/2024 |
| 0.2 | nonfan | 1440 | unknown | 9/23/2024 |
| 1.3 | nonfan | 1446 | unknown | 9/24/2024 |
| 2.1 | fan | 1460 | unknown | 9/26/2024 |
| 2.2 | nonfan | 1463 | 14 days | 9/26/2024 |
| 2.2 | fan | 1472 | 14 days | 9/26/2024 |
| 1.1 | fan | 1476 | unknown | 9/24/2024 |
| 1.3 | nonfan | 1492 | unknown | 9/24/2024 |
| 1.1 | fan | 1493 | unknown | 9/24/2024 |
| 1.1 | fan | 1495 | unknown | 9/24/2024 |
| 2.1 | nonfan | 1500 | 14 days | 9/26/2024 |
| 0.2 | fan | 1500 | unknown | 9/23/2024 |
| 0.2 | fan | 1500 | unknown | 9/23/2024 |
| 2.1 | nonfan | 1536 | unknown | 9/26/2024 |
| 1.3 | nonfan | 1555 | unknown | 9/24/2024 |
| 2.1 | nonfan | 1559 | 14 days | 9/26/2024 |
| 0.2 | nonfan | 1560 | unknown | 9/23/2024 |
| 1.3 | fan | 1577 | unknown | 9/24/2024 |
| 1.2 | fan | 1578 | unknown | 9/24/2024 |
| 1.2 | nonfan | 1620 | unknown | 9/24/2024 |
| 1.3 | nonfan | 1632 | unknown | 9/24/2024 |
| 2.2 | nonfan | 1640 | 14 days | 9/26/2024 |
| 1.3 | fan | 1670 | unknown | 9/24/2024 |
| 1.2 | fan | 1674 | unknown | 9/24/2024 |
| 1.3 | nonfan | 1683 | unknown | 9/24/2024 |
| 1.1 | fan | 1693 | unknown | 9/24/2024 |
| 1.3 | fan | 1696 | unknown | 9/24/2024 |
| 1.3 | nonfan | 1714 | unknown | 9/24/2024 |
| 1.2 | fan | 1733 | unknown | 9/24/2024 |
| 0.2 | fan | 1740 | unknown | 9/23/2024 |
| 2.2 | nonfan | 1792 | unknown | 9/26/2024 |
| 0.1 | nonfan | 1800 | unknown | 9/23/2024 |
| 0.2 | nonfan | 1800 | unknown | 9/23/2024 |
| 0.2 | nonfan | 1800 | unknown | 9/23/2024 |
| 1.3 | fan | 1855 | unknown | 9/24/2024 |
| 0.1 | nonfan | 1860 | unknown | 9/23/2024 |
| 2.2 | nonfan | 1912 | 14 days | 9/26/2024 |
| 0.1 | nonfan | 1920 | unknown | 9/23/2024 |
| 1.1 | nonfan | 1939 | unknown | 9/24/2024 |
| 0.2 | fan | 1980 | unknown | 9/23/2024 |
| 0.1 | nonfan | 1980 | unknown | 9/23/2024 |
| 1.2 | nonfan | 1990 | unknown | 9/24/2024 |
| 1.2 | nonfan | 2011 | unknown | 9/24/2024 |
| 1.1 | nonfan | 2019 | unknown | 9/24/2024 |
| 0.2 | fan | 2040 | unknown | 9/23/2024 |
| 2.2 | nonfan | 2057 | 14 days | 9/26/2024 |
| 1.3 | fan | 2154 | unknown | 9/24/2024 |
| 1.2 | fan | 2228 | unknown | 9/24/2024 |
| 2.1 | nonfan | 2264 | 14 days | 9/26/2024 |
| 1.2 | fan | 2279 | unknown | 9/24/2024 |
| 1.2 | nonfan | 2293 | unknown | 9/24/2024 |
| 2.1 | fan | 2315 | 14 days | 9/26/2024 |
| 1.1 | nonfan | 2402 | unknown | 9/24/2024 |
| 1.1 | fan | 2519 | unknown | 9/24/2024 |
| 0.1 | nonfan | 2520 | unknown | 9/23/2024 |
| 1.1 | fan | 2585 | unknown | 9/24/2024 |
| 1.2 | nonfan | 2806 | unknown | 9/24/2024 |
